# The origin of Rosenthal fibers and their contributions to astrocyte pathology in Alexander disease

**DOI:** 10.1186/s40478-017-0425-9

**Published:** 2017-03-31

**Authors:** Alexander A. Sosunov, Guy M. McKhann, James E. Goldman

**Affiliations:** 1 Department of Neurosurgery, Columbia University, New York, NY 10032 USA; 2 Department of Pathology and Cell Biology, Columbia University, New York, NY 10032 USA

**Keywords:** Rosenthal fibers, Alexander disease, Astrocytes, GFAP, alphaB-crystallin

## Abstract

Rosenthal fibers (RFs) are cytoplasmic, proteinaceous aggregates. They are the pathognomonic feature of the astrocyte pathology in Alexander Disease (AxD), a neurodegenerative disorder caused by heterozygous mutations in the *GFAP* gene, encoding glial fibrillary acidic protein (GFAP). Although RFs have been known for many years their origin and significance remain elusive issues. We have used mouse models of AxD based on the overexpression of human *GFAP* (transgenic, TG) and a point mutation in mouse *GFAP* (knock-in, KI) to examine the formation of RFs and to find astrocyte changes that correlate with the appearance of RFs. We found RFs of various sizes and shapes. The smallest ones appear as granular depositions on intermediate filaments. These contain GFAP and the small heat shock protein, alphaB-crystallin. Their aggregation appears to give rise to large RFs. The appearance of new RFs and the growth of previously formed RFs occur over time. We determined that DAPI is a reliable marker of RFs and in parallel with Fluoro-Jade B (FJB) staining defined a high variability in the appearance of RFs, even in neighboring astrocytes. Although many astrocytes in AxD with increased levels of GFAP and with or without RFs change their phenotype, only some cells with large numbers of RFs show a profound reconstruction of cellular processes, with a loss of fine distal processes and the appearance of large, lobulated nuclei, likely due to arrested mitosis. We conclude that 1) RFs appear to originate as small, osmiophilic masses containing both GFAP and alphaB-crystallin deposited on bundles of intermediate filaments. 2) RFs continue to form within AxD astrocytes over time. 3) DAPI is a reliable marker for RFs and can be used with immunolabeling. 4) RFs appear to interfere with the successful completion of astrocyte mitosis and cell division.

**Electronic supplementary material:**

The online version of this article (doi:10.1186/s40478-017-0425-9) contains supplementary material, which is available to authorized users.

## Introduction

Rosenthal fibers (RFs) are distinctly characteristic features of the astrocyte pathology in Alexander Disease (AxD), a neurodegenerative disorder caused by heterozygous mutations in the gene encoding glial fibrillary acidic protein (GFAP), the major intermediate filament in astrocytes [15, 21]. RFs are protein aggregates in the cytoplasm of astrocytes. They were originally identified by inspection with the light microscope as bright, eosinophilic profiles inside astrocytes. Ultrastructurally, RFs appear as amorphous, electron dense material, surrounded by skeins of intermediate filaments, and are not membrane bound [1, 10, 19, 23]. RFs are composed mainly of intermediate filaments, GFAP [36], vimentin, and synemin [20] in addition to other proteins including the small heat shock proteins alphaB-crystallin [36] and Hsp 27 [5], the filament binding protein plectin [35], and Cyclin D2 [9].

Although RFs are characteristic of AxD, they are not unique to this disease. For example, RFs are regularly found in pilocytic astrocytomas and chronic astrocyte scars [4, 11, 26]. Occasionally astrocytes in other disorders, such as cortical dysplasias and tuberous sclerosis, contain RFs [13, 14]. Presumably the common denominator is the accumulation of very large amounts of GFAP inside the astrocyte [16]. RFs vary substantially in size, from very large to small, the latter appearing at the light microscope level as a cytoplasmic, eosinophilic granularity.

Many neuropathologists have shown electron micrographs of RFs, but there is still little known about the formation of these inclusions. To gain insights into this question, we analyzed RFs during the progression of AxD in mouse models. We have used four murine models of AxD: 1) one based on the insertion of additional copies of human *GFAP* (transgenic AxD mice, TG); 2) one based on replacement of one copy of *GFAP* with a mutant (R236H mutation, the mouse homolog of the most common human AxD mutation) mouse *GFAP* (heterozygous knock-in AxD mice, heterozygous KI); 3) homozygous knock-in (KI) AxD mice, with replacement of both copies of GFAP with R236H mutant mouse GFAP; 4) the offspring of transgenic (TG) and homozygous knock-in (KI) mice (double mutant) [7, 16]. The latter are severely affected, dying within 5 weeks with seizures [7]. All of these mice are characterized by an abundance of RFs. We performed electron microscopic examinations of RFs and found evidence that RFs form from small accumulations of electron dense material, containing GFAP and alphaB-crystallin, on intermediate filaments. These small RFs appear to merge with the edges of larger RFs. We also found that in addition to Fluoro-Jade B that DAPI staining is a sensitive marker of RFs, one that can be combined with immunofluorescence.

## Material and methods

### Mice

The TG and KI AxD murine models have been introduced and previous described [5, 15]. These mice could survive for more than 1 year without features of illness or fatigue. Cross breeding of TG and KI produce mice that survive about 1 month and die after severe seizures. The TG line was initially generated in an FVB background, but the mice were crossed into a B6 background over at least five generations before they were used for these experiments. The KI line was initially generated in mice with a B6 background but now is maintained on a mixed 129S6 x FVB/N strain. Only male mice were used in the experiments. In total we used 9 double mutant (3 at the age of 1 week and 6 at the age of 1 month), 10 TG (4 at the age of 1 month and 6 at the age of 1 year), and 14 KI (homozygous, 2 at the age of 1 month and 5 at the age of 1 year; heterozygous, 2 at the age of 1 month and 4 at the age of 1 year). All animal use was performed under the guidelines of the Columbia University Institutional Animal Care and Use Committee.

### Human subjects

Autopsy tissue (fixed in formalin and embedded in paraffin) from two (F, 11 month of age and F, 3 years old) AxD patients was used for analysis. Diagnosis was confirmed by histopathological examination and genetic analysis for *GFAP* mutations. Postmortem intervals were less than 10 h.

### Histology and immunohistochemistry

Mice were anesthetized with ketamine-xylazine before intracardiac perfusion with 4% paraformaldehyde in PBS. Brains were removed and kept in the fixative for 12 - 16 h (4^0^ C). Forty μm coronal sections were prepared with a vibratome (Leica VT1000S) and stored in cryoprotectant solution at -20^0^ C before use. Paraffin sections from the blocks of autopsy AxD human brains were deparaffinized and were used for immunostaining. Some were stained with H&E.

Primary antibodies were used against: (i) glial fibrillary acidic protein (GFAP): mouse monoclonal (1:1000, G3893, Sigma-Aldrich, St. Louis, MO), rabbit polyclonal (1:1000, Z 0334, Dako, Carpinteria, CA); (ii) CD44: rat monoclonal (1:200, #217594, Millipore); (iii) Ki67: mouse monoclonal (1:100, #550609, BD Pharmingen, San Jose, CA); (iv) a centrosome marker, pericentrin: rabbit polyclonal (1:400, PRB-432C, Covance). Secondary antibodies included: anti-mouse Alexa Fluor 488 and 594; anti-rabbit Alexa Fluor 488 and 594; anti-rat Alexa Fluor 488 and 594; all from goat or donkey (1:300, Molecular Probes, Eugene, OR).

For immunofluorescence, after blocking with 10% normal goat (or donkey) serum (30 min, at room temperature (RT)), free-floating sections were incubated in a mixture of primary antibodies raised in different species for overnight (4^0^ C). For visualization, Alexa Fluor-conjugated secondary antibodies were applied for 1 h at RT. For visualization of nuclei Fluorescent Nissl reagent (NeuroTrace 640/660 deep-red, 1:150, Molecular Probes, 30 min after secondary antibodies, RT) and DAPI (5 μg/ml; D9542, Sigma-Aldrich, added to secondary antibodies for the last 10 min of incubation) were used. Paraffin sections and mouse brain sections were treated with Antigen Unmasking Solution (Vector Laboratories, # H-3300, Burlingame, CA) according to manufacturer’s recommendations before blocking with serum. Blocking serum, primary, secondary antibodies, and DAPI were applied in 0.2% Triton X-100 in PBS. Sections for fluorescent microscopy were mounted on slides in Vectashield (Vector Lab). To control for the specificity of immunostaining, primary antibodies were omitted and substituted with appropriate normal serum. Slides were viewed using a Nikon A1R MP confocal microscope. 3D reconstructions were generated from stacks of images with confocal microscope software NIS-Elements.

***Fluoro-Jade B Staining*** was performed according to manufacturer’s recommendations (Millipore). Briefly, 40 μm coronal brain slices were mounted on the slides, dried, exposed to 1% NaOH in 80% alcohol for 5 min, followed by 70% alcohol (2 min) and distilled water (2 min). After pretreatment in 0.006% potassium permanganate (15 min RT) followed by washing (distilled water, 2 min), slides were incubated in 0.0004% of FluoroJade B (in distilled water with 0.1% acetic acid) for 20 min (RT) and after drying were coverslipped with DPX (Sigma-Aldrich). This method was initially proposed and is widely used for the detection of degenerating neurons [22]. Reactive astrocytes [3] and Rosenthal fibers [30] were also stained with Fluoro Jade B.

### Electron microscopy (EM)

For regular transmission EM vibratome slices were additionally fixed in 2.5% glutaraldehyde in PBS (several hrs at 4 °C), postfixed in 2% osmium tetroxide in 0.2 PBS (2 h at 4 °C), dehydrated and flat-embedded in Epon-Araldite (Electron Microscopy Sciences, Hatfield, PA) on ACLAR Embedding Film (Ted Pella, Inc., Redding, CA). Areas of interest were identified under light microscope, cut from sections, and glued onto resin blocks. Ultrathin sections were cut with Reichert Ultracut E, stained with uranyl acetate and lead citrate, and examined with a JEOL 1200 electron microscope.

For postembedding immunogold immunocytochemistry small pieces of the neocortex and hippocampus were embedded in LR White Resin (Berkshire, England). Ultrathin sections on nickel grids were blocked with 10% donkey serum (30 min, RT), incubated with primary antibodies anti-GFAP (rabbit polyclonal, 1:200, Dako, for single staining, and mouse monoclonal, Sigma, 1:100), for double immunostaining with anti-alphaB-crystallin (rabbit polyclonal, 1:100, Millipore) applied overnight at 4^0^ C, followed by secondary antibodies conjugated with gold particles (12 nm in diameter for GFAP, and 18 nm for alphaB-crystallin) (all at 1:50) for 1.5 h at RT. Blocking donkey serum, primary and secondary antibodies were applied in PBS and grids were put on the top of droplets of corresponding solutions. Ultrathin sections were stained with uranyl acetate and examined with a JEOL 1200 electron microscope.

### Quantitative analysis

The numbers of RFs and the area they occupied were counted in the images (merged from stacks of 6 adjacent images with 1024 × 1024 pixel resolution, observed area 295 × 295 μm, captured by confocal microscopy at a distance of 0.5 μm from each other) obtained from neocortices in coronal sections stained with Fluoro-Jade B (10 images from each section, 5 sections per animal). Images were transferred to Image J 1.46r (public domain), grayscaled and quantified based on the optical density (OD). The size of small RFs, detected with the electron microscope, and the numbers of RFs with different shapes were determined in EM images. We counted RFs in all parts of astrocytes, including cell bodies, processes, and endfeet.

### Statistical analysis

Data were expressed as mean ± SEM. Student’s *t*-test was used as appropriate for parametric data. For non-parametric data Chi-square test was used. *P* <0.05 was considered significant.

## Results

### RFs appear to form from small aggregates

**Fig. 1:**
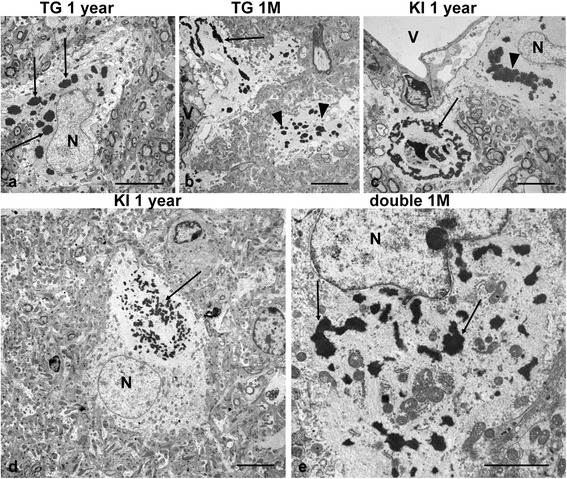
Variability in the ultrastructure of Rosenthal fibers (RFs) in AxD mice. **a** RFs with oval shape (*arrows*, only some marked) in the astrocyte body and proximal part of the process. TG 1 year-old mouse. **b** Elongated RFs (*arrow*) in the astrocyte endfoot near blood vessel (V), note that in neighboring astrocyte process RFs have oval shape (*arrowheads*). TG 1 month-old mouse. **c** Different morphologies of RFs in neighboring astrocytes near blood vessel (V). Note that in lower left group of RFs (*arrow*) dark central material is encircled by small undulating, ramified bands of RFs, whereas in other cell oval shape RFs form compact group (*arrowhead*). KI homozygous 1 year-old mouse. **d, e** Focal (**d**) and dispersed (**e**) localizations of RFs in the cytoplasm. Note that in (**d**) the area with RFs and surrounding filaments (*arrow*) is sharply demarcated from cytoplasm whereas in (**e**) RFs (*arrows*) are distributed within cytoplasm and intermingled with organelles. **d** KI homozygous 1 year-old mouse. **e** double mutant 1 month-old mice. N- astrocyte nucleus. Scale bars: 7 μm in (**a**)-(**d**); 4 μm in (**e**)

Based upon the many studies of RF ultrastructure, we defined RFs as non-membrane bound, cytoplasmic, electron-dense material directly linked to intermediate filaments. We found a high degree of variability in the shapes and sizes of RFs in each AxD line (Fig. 1). RFs were observed in every part of astrocyte cell bodies and processes, although predominantly in perikarya and in perivascular and subpial endfeet, which were enlarged due to the accumulation of filaments and RFs (Fig. 1a, b, c). In some cell bodies RFs with surrounding filaments were clearly and sharply segregated from the surrounding cytoplasm (Fig. 1d). In other cells, RFs with neighboring filaments were distributed in the cytoplasm and intermingled with cytoplasmic organelles (Fig. 1e).

**Fig. 2:**
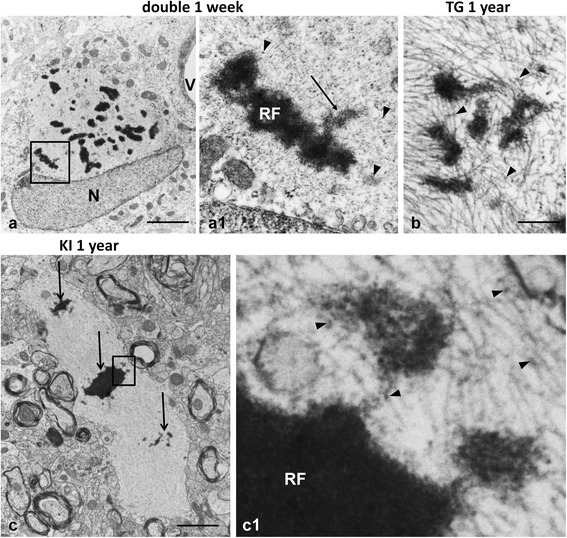
Small RFs in AxD mice. **a** RFs in the cell body of astrocyte near blood vessel (V). double mutant 1 week-old mouse. **a1** enlarged boxed area in (**a**). Note a small osmiophilic protrusion (*arrow*) from Rosenthal fiber (RF) composed of granular material and profiles of filaments. Granular material, similar in size to those in the inclusions, are attached to filaments or from small groupings (*arrowheads*). **b** A group of small, electron dense bodies connected by filaments in TG 1 year-old mouse. *Arrowheads* indicate small aggregates on filaments. **c** Astrocyte process with RFs (*arrows*) of different sizes. KI homozygous 1 year-old mouse. **c1** enlarged boxed area in (**c**). Note that two small osmiophilic profiles are ‘connected’ to large Rosenthal fiber (RF) by granular, electron dense material located on filaments. The electron dense material of small profiles has a granular pattern and includes filaments. Electron dense granules (*arrowheads*) similar in size to those in the aggregates are located on filaments around the RF. N –nucleus of the astrocyte. Scale bars: 6 μm in (**a**) and (**c**); 250 nm in (**b**)

Small, dark accumulations of moderate electron density (average size 233 ± 8 nm, range 38–410 nm) were often situated near RFs (Fig. 2a and c) and connected to them by intermediate filaments or thin zones of electron dense material (Fig. 2a1, b and c). We believe these small electron dense profiles are also RFs. The electron density of the small RFs was lower and more heterogeneous than that of large RFs and it was possible to visualize intermediate filaments running through the small profiles (Fig. 2a1, b and c1), indicating that filaments were preserved and likely represented a “backbone” on which material deposited. A close look at the small RFs also revealed what appeared to be many small, granular profiles within the matrix of the inclusion (Fig. 2a1, b and c1). This granular material was the same size as that observed on individual filaments outside of the RF itself (Fig. 2c1). These small profiles may represent precipitation of proteins on filaments that could build and eventually lead to small RF formation (see Discussion). The large RFs looked more homogeneous and did not display visible filaments within the matrix (Figs. 2a1 and 3). In many cases profiles with high electron density were surrounded or ‘combined’ with less dense and less homogeneous material, thus producing a high variability in the appearance of RFs (Figs. 2a1 and 3a1).

**Fig. 3:**
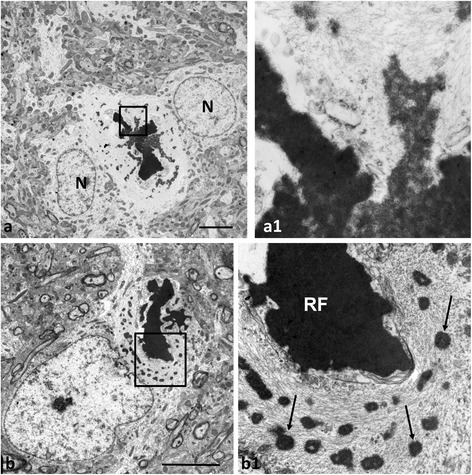
Variability in the electron dense material of RFs. **a** Large RF is composed of two types of materials, one homogeneously dark and other with moderate electron density Note that astrocyte has two nuclear profiles (N). **a1** Enlarged boxed region in (**a**), showing heterogeneity of RFs. **b** Central large RF is surrounded by many small RFs. **b1**) enlarged boxed area in (**b**). Note that large RF (RF) is encircled by membranes. Small neighboring RFs (*arrows*, only some marked) are not membrane-bound. KI homozygous 1 year-old mice. Scale bars: 5 μm

We found that some of the large profiles of electron-dense material were enclosed by double membranes, suggesting that these were enfolded by autophagic vacuoles (Fig. 3b). It was only the homogeneous material with high electron density that was surrounded by membranes.

**Fig. 4:**
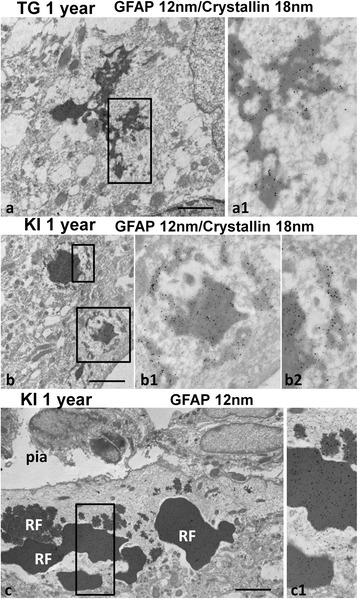
Immunogold staining of RFs. **a**, **b** Double immunogold staining for alphaB-crystallin (18 nm particles) and GFAP (12 nm particles) in 1 year-old TG (**a**) and KI homozygous (**b**) mice. Note that every large and small electron dense profile of RFs contains small (GFAP) and large (alphaB-crystallin) gold particles indicating the presence of both antigens. **c**) Immunogold staining of RFs in subpial astrocytes for GFAP (12 nm particles). Note that large and small electron dense profiles are diffusely covered with gold particles. KI homozygous 1 year old mouse. **a1**, **b1**, **b2**, and (**c1**)–enlargements of boxed areas in (**a)**, (**b)**, and (**c**) respectively. Scale bars: 1 μm in (**a**),(**c**); 2 μm in (**b**)

GFAP and alphaB-crystallin are both major components of RFs [36]. We performed post-embedding immunogold electron microscopy and as expected, the large RFs were labeled with both antibodies (Fig. 4a and b). We paid particular attention to the small RFs and found both proteins associated with the small, less dense inclusions (Fig. 4a and b), suggesting that both of these proteins form components of the earliest RFs.

### Variability in the shapes and sizes of RFs

The variability in the ultrastructure of RFs was a characteristic feature of all 4 types of murine AxD models and many forms of RFs could be found in each line, although some forms of RFs might be considered characteristic. We paid special attention to comparing the TG and KI mice because these lines clearly represent different alterations in GFAP composition. Thus, RFs shown in Fig. 1c were observed only in KI mice. RFs in form of undulated ramified bands of electron dense material shown in Fig.1c and Additional file 1: Figure S1C predominated in KI mice and constituted 54% of all RFs in KI homozygous mice vs 20% in TG mice (*P* <0.001, Chi-Square test).

Despite the high degree of variability of shapes and sizes of RFs, it was possible to discriminate three main types: 1) large RFs with an oval to round shape and a size more than 0.4 μm in diameter/width (Fig. 1a, c, e; Additional file 1: Figure S1b, D), 2) elongated RFs (several μm in length and with widths of 0.3–0.5 μm) (Fig. 1b), 3) small RFs (less than 0.4 μm in width) with puncta-like (Fig. 2a, b and c; Additional file 2: Figure S4c, astrocyte 2) or ramified, ribbon-like (Fig. 1c, Additional file 1: Figure S1c) shapes. Combinations of these types could be present even in one astrocyte and neighboring astrocytes often differed in the size and shape of RFs. The maximal size of RFs appeared to be limited and did not surpass ~ 9 μm in the long axis.

### DAPI is a reliable marker for RFs

**Fig. 5:**
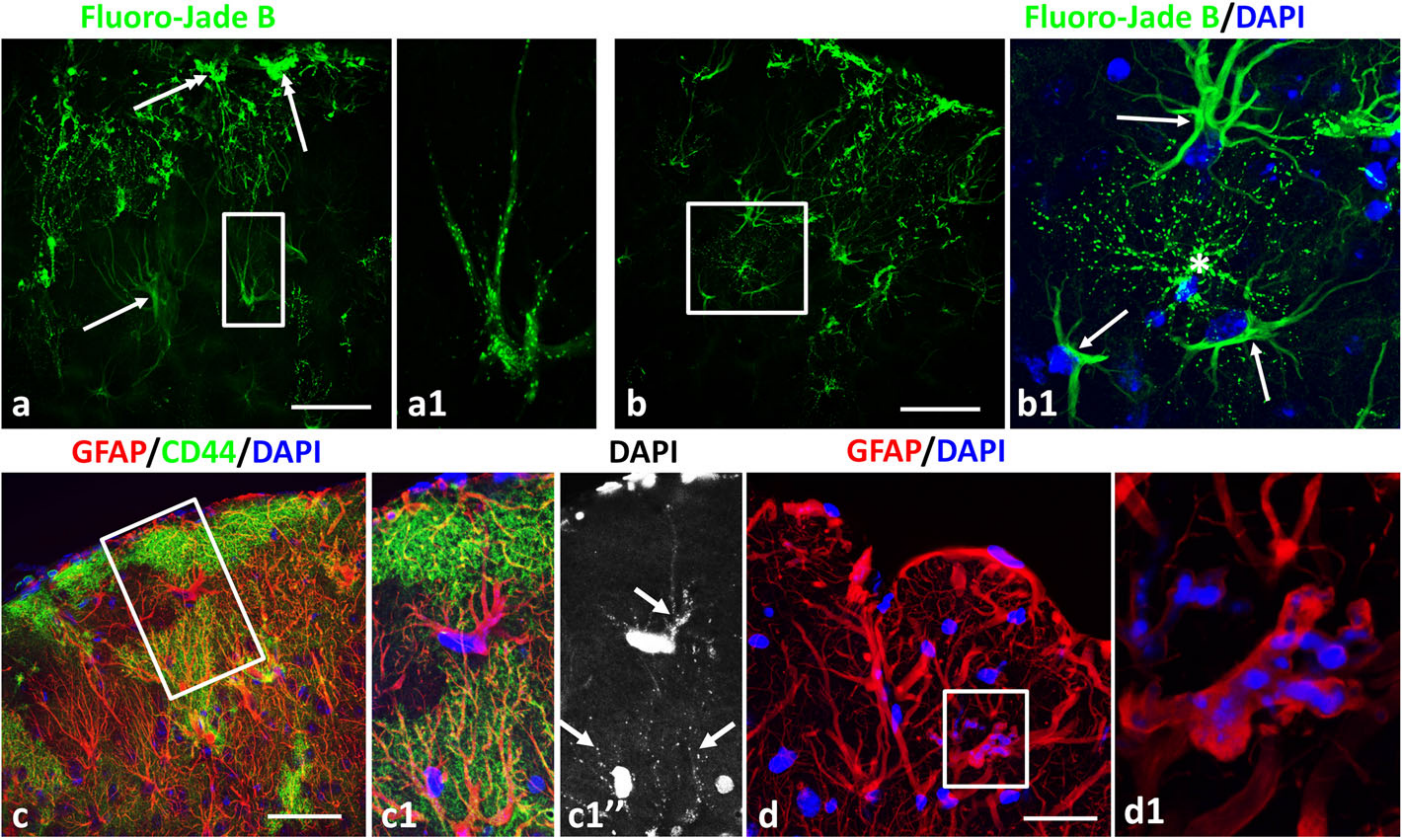
RFs visualized with Fluoro-Jade B (FJB) (**a**, **b**) and DAPI combined with immunohistochemical staining (**c**-**d**) in 1 year-old TG mice. **a** Subpial area rich in FJB+ RFs (bright green profiles). Note large size of RF aggregates in the vicinity of the pia (double headed *arrows*). *Arrow* indicates astrocyte without RFs visualized with FJB in cell body and processes. **a1** Enlarged boxed area in (**a**) shows an astrocyte with many RFs in the form of small, bright puncta. **b** Variability of astrocytes in subpial area. In (**b1**) (enlarged boxed area in **b**), with DAPI counterstaining, which stains nuclei but not RFs, in combination with FJB. The astrocyte with many RFs (*asterisk*) is surrounded by astrocytes (arrows) without RFs. **c** Subpial area with a high level of astrogliosis. Double immunostaining for GFAP and CD44, counterstaining with DAPI. Confocal microscopy. **c1** Enlarged boxed area in (**c**). Note: 1) small bright profiles (*arrows* in (**c1**)”, marked only some) in astrocyte bodies and processes corresponded to RFs stained with DAPI, 2) Top astrocyte (in **c1**) does not stain for CD44, but bottom left astrocyte does. Both contain many RFs. **d**) Aggregates of RFs in astrocyte processes near the pial surface. **d1** –enlarged boxed area in (**d**). Large irregular profile of astrocyte process filled with many RFs that reveal minimal GFAP immunostaining of the central part. For animation of Z-stack of optical slices in image (**d1**) see Additional files 5 and 6: Movie 1 and 1a. Immunostaining for GFAP, counterstaining with DAPI. Black and white image show DAPI staining. Confocal microscopy. Scale bars: 60 μm in (**a**)-(**c**); 25 μm in (**d**)

**Fig. 6:**
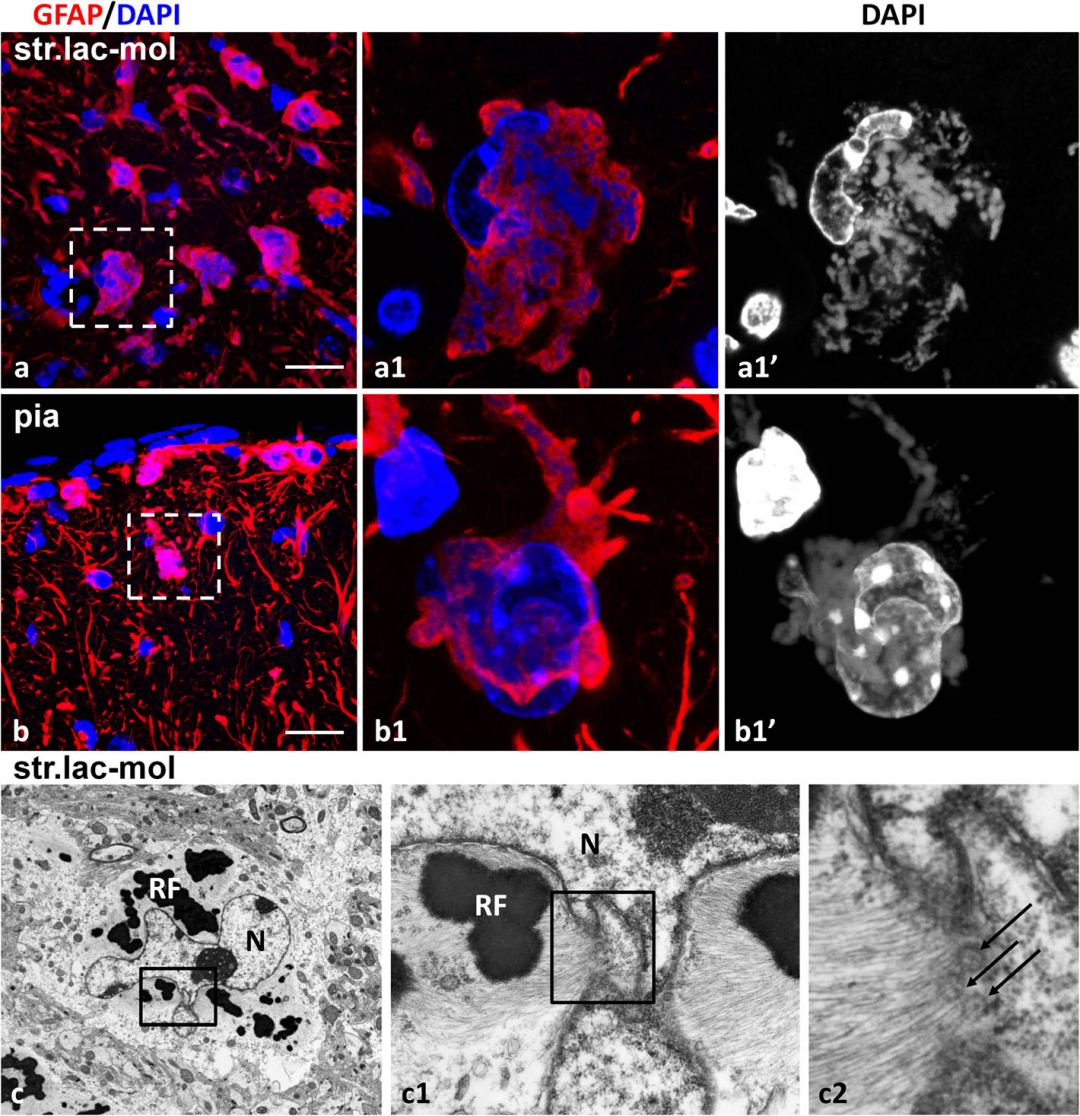
Aberrant types of astrocytes in double mutant 1 month old. **a** Abnormal astrocytes in str. lacunosum-moleculare (str.lac-mol) with irregular shape and abundance of variably shaped and sized RFs. **a1** Enlarged boxed area in (**a**) shows astrocyte with a lobulated nucleus (An animation of Z-stack of optical slices in image (**a1**)’ see in Additional file 7: Movie 2) and many polymorphic Rosenthal fibers (in black and white in (**a1**)’). **b** Abnormal astrocytes in the subpial area. **b1** Enlarged boxed area in (**b**) shows an astrocyte with a lobulated nucleus and many RFs. An animation of Z-stack of optical slices in image (**b1**)’ is shown in Additional file 8: Movie 3. **c** Ultrastructure of an astrocyte in the str. lacunosum-moleculare (str.lac-mol) with a lobulated nucleus (N) and RFs. (**c1**) Enlarged boxed area in (**c**). Note RF with surrounding intermediate filaments near a protrusion of the nucleus. **c2**) Enlarged boxed area in (**c1**). Note that intermediate filaments are in close apposition to nuclear pores (arrows). **a** and **b** –confocal images from slices stained for GFAP and counterstained with DAPI. Black and white images show DAPI staining. Scale bars: 55 μm in (**a**), 85 μm in (**b**), and 2 μm in (**c**)

To examine the distribution and variability of RFs further we used Fluoro-Jade B staining (FJB), a marker of RFs [30] (Fig. 5a and b). We also found that DAPI, usually used for the visualization of nuclei, regularly stained small puncta in astrocytes that corresponded in size and location to RFs (Fig. 5c and d; Fig. 6a and b). We could not get reproducible and reliable double staining of RFs with FJB and DAPI together. Therefore, we examined RFs in homozygous KI mice where many RFs are clearly distinguished with GFAP immunolabeling (Additional file 3: Figure S2a, d). In KI homozygous mice every profile of high GFAP immunostaining was co-localized with DAPI+ material (Additional file 4: Figure S3a, a1). We examined two autopsy specimens of human AxD patients and also found that every GFAP immunopositive profile that corresponded to a RF was also stained with DAPI (Additional file 4: Figure S3b). Thus, we concluded that DAPI is a reliable marker of RFs and used it in parallel with, but not together with, FJB for analysis of RFs.

Staining with FJB and DAPI revealed that RFs predominated in the areas with high levels of astrogliosis and in the neocortex they were found in abundance in upper cortical layers (Fig. 5). The largest numbers of RFs were found in the double mutant and the TG mice. The central DAPI+ part of RFs was usually GFAP immunonegative (Figs. 5d; 6a1 and b1). In ultrathin sections with immunogold post-embedding labeling, the osmiophilic material of every large RF examined near the pia and near blood vessels was heavily labeled with gold particles indicating the presence of GFAP (Fig. 4c). Such a discrepancy in immunostaining at the light and electron microscopic levels may be explained by absorption of anti-GFAP antibodies by densely packed GFAP filaments surrounding RFs and the inability of antibodies to penetrate into the dense central matrix of RFs. Antigens are far more accessible in the ultrathin sections used in electron microscopy [36].

In line with previous studies [2], we observed that FJB stained cell bodies and processes of reactive astrocytes filled with GFAP. Such properties of FJB staining, in parallel with DAPI staining, allowed us to show that astrocytes with RFs were often found in neighboring astrocytes that were filled with GFAP but did not contain RFs (Fig. 6a and b; Additional file 2: Figure S4b). It is worth noting that the resolution of confocal microscopy and the staining properties of FJB and DAPI may not allow us to visualize the smallest RF seen in the electron microscope. But even considering this possibility the difference between neighboring astrocytes is striking. Variability in RF shapes and size was regularly observed, and even adjacent astrocytes often differed in the shapes and sizes of RFs (Fig. 1c, Additional files 5 and 6: Movie 1 and 1a, Additional file 2: Figure S4b, c).


Additional file 5: Movie 1. Animation of Z stack of optical slices of Fig. 5d1, GFAP and DAPI double stained. (AVI 1509 kb)



Additional file 6: Movie 1. Animation of Z stack of optical slices of Fig. 5d1, DAPI double stained only. (AVI 1509 kb)


### RFs accumulate and enlarge with age

To assess the appearance of RFs with age, we compared RFs in 1 month and 1 year old TG and KI mouse cortex with FJB and found a significant increase in the numbers (for TG: 159.9 ± 17.8 RFs in 1 month old vs 406.9 ± 48.7 in 1 year old mice per 12,155 μm^2^, *P* = 0.005; for KI: 65.4 ± 15.6 in 1 month old vs 186.0 ± 33.7 in 1 year old mice per 12,155 μm^2^, *P* = 0.008) and sizes of RFs (in TG in 1 month old mice the average size of RFs was 3.03 ± 0.6 μm^2^ vs 41.9 ± 2.8 μm^2^ in 1 year old mice *P* = 0.008 and in KI in 1 month old mice the average size of RFs was 2.25 ± 0.2 μm^2^ vs 3.36 ± 0.26 μm^2^ in 1 year old mice *P* = 0.008), indicating the continuous formation and enlargement of RFs with time in both lines of mice. These data are in line with previous results [17] that TG mice reveal more severe pathology compared to KI mice.

### Accumulation of RFs correlates with changes in astrocyte phenotype

**Fig. 7:**
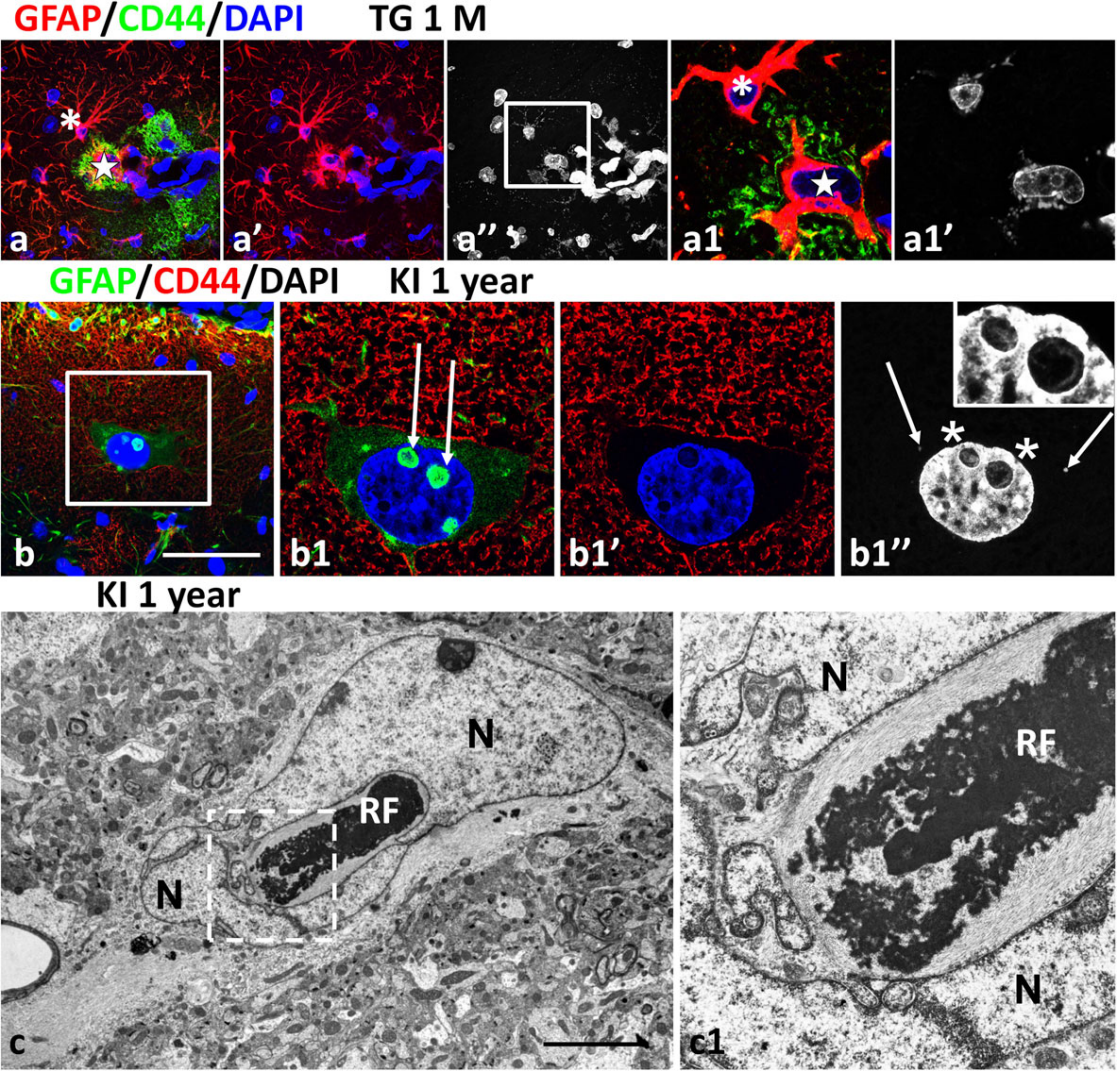
Phenotypic alterations in astrocytes with many RFs. **a** Neocortical CD44+ astrocyte (star) with thickened, short main processes filled with RFs lacking miniature peripheral processes (DAPI stains in (**a**)” and (**a1**)’). Note that neighboring astrocyte (*asterisk*) with many RFs (DAPI stains in (**a)**” and (**a1**)’**)** does not show immunoreactivity for CD44. **a1** Single optical slice from enlarged boxed area in (**a**)**”**. Animation of Z-stack of optical slices of (**a1**) see in Additional file 9: Movies 4. 1 month old TG mouse. Double immunostaining for GFAP and CD44. Counterstaining with DAPI. Confocal microscopy. **b** Large astrocyte in cortical layer I with GFAP+ pseudoinclusions in the nucleus (*arrows* in (**b1)**) corresponding to RFs located in deep invaginations of nuclear envelope (inset in (**b1**)” shows enlarged area of the nucleus marked with *asterisks*). Note the large size of the nucleus and only few Rosenthal fibers (RFs) (*arrows* in **b1**”) in the cell body near nucleus. **b1**’ Note also that CD44 outlined densely packed miniature distal processes of the astrocyte. **b1** enlarged boxed area in (**b**). KI homozygous 1 year old mouse. Double immunostaining for GFAP and CD44. Counterstaining with DAPI. Confocal microscopy. **c** Ultrastructure of astrocyte with Rosenthal fibers (RFs) in deep invagination of the nucleus (N). Note irregular shape of the nucleus. KI homozygous 1 year old mice. Scale bars: 60 μm in (**a**) and (**b**); 0.6 μm in (**c**)


Previously we reported abnormal astrocyte phenotypes in double mutant mice (see Fig. 5 in [28]). These cells showed shortened processes ending with bulbous enlargements (in contrast to the tapered processes of normal astrocytes) and a reduction in the (normal) miniature, distal processes. Another characteristic feature of such astrocytes was a highly lobulated nucleus or several micronuclei. We examined whether the presence of RFs correlated with these changes. Indeed, every aberrant astrocyte (79 cells with abnormal processes and nuclei examined in hippocampus and cortex) in the double mutant mice contained an abundance of RFs (Fig. 6a and b; Additional files 7 and 8: Movie 2 and 3). Many RFs with surrounding filaments were in close apposition to the nuclear envelope (Fig. 6c). Similarly aberrant astrocytes filled with RFs were found in TG mice. They had abnormal shapes of main processes and reduced numbers of small distal processes (as was visualized with CD44 immunostaining of the plasma membrane) (Fig. 7a; Additional file 9: Movie 4).

Additional file 7: Movie 2. Animation of Z stack of optical slices of Fig. 6a1’. (AVI 2769 kb)



Additional file 8: Movie 3. Animation of Z stack of optical slices of Fig. 6c1’. (AVI 612 kb)



Additional file 9: Movie 4. Animation of Z stack of optical slices in Fig. 7a1. (AVI 7408 kb)


In KI mice there were many enlarged astrocytes with large, lobulated nuclei but the cells did not display the abnormalities in process geometry seen in the TG and double mutant mice, but rather preserved their miniature, distal processes, shown with CD44 immunostaining (Fig. 7b and b1). However, these astrocytes usually had only a few RFs in the cytoplasm of cell bodies and processes (Fig. 7b1”), in contrast to the larger numbers of RFs in astrocytes in the TG or double mutant mice.

**Fig. 8:**
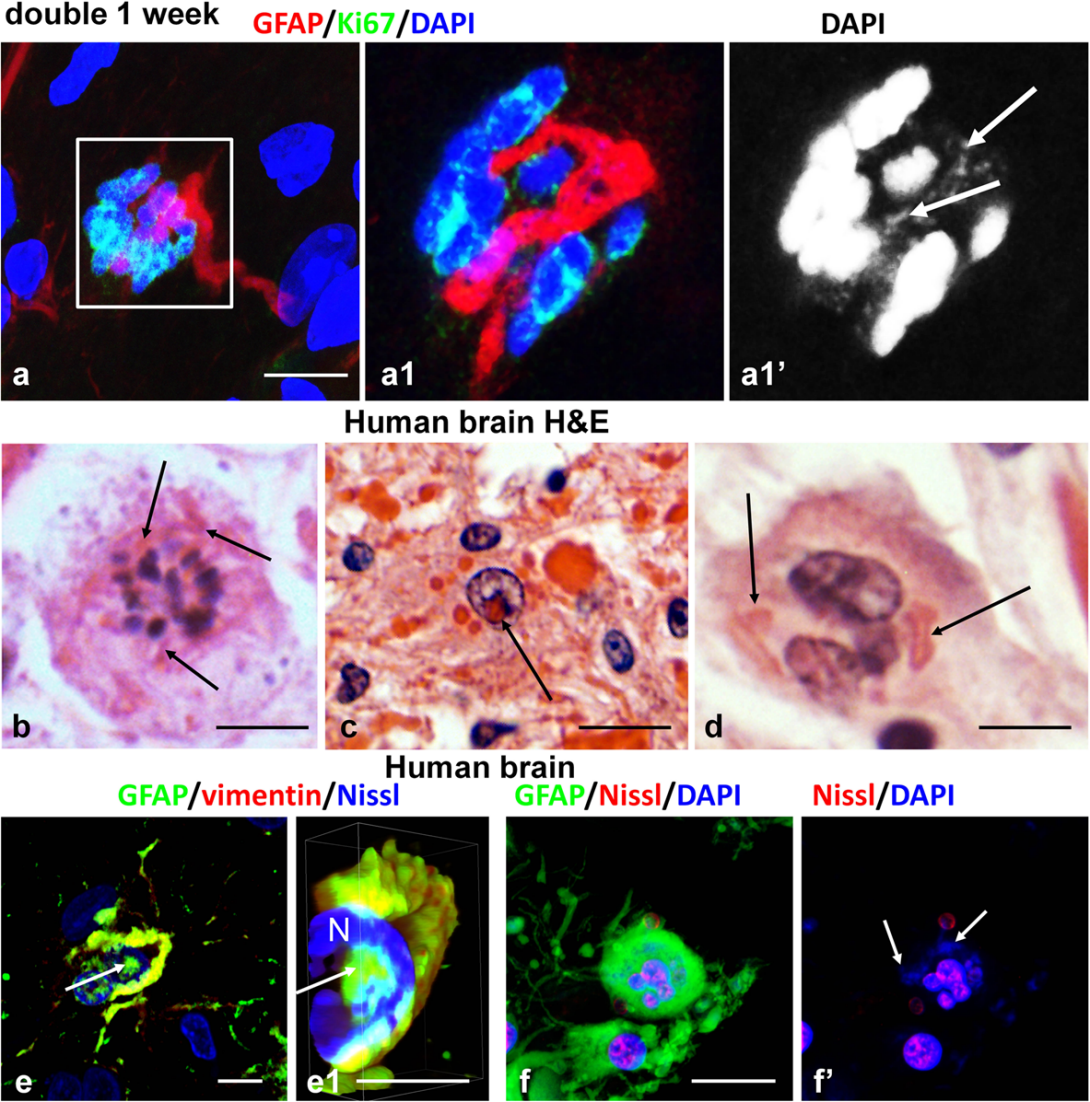
Mitotic abnormalities in astrocytes with Rosenthal fibers (RFs) in AxD mouse (**a**) and AxD human brains (**b**-**f**). **a** RFs (*arrows* in (**a1**)’) between metaphase chromosomes in a mitotic astrocyte in 1 week-old double mutant mouse. **A1** Single optical slice of enlarged boxed area in (**a**). Double immunostaining for GFAP and Ki67, counterstaining with DAPI. Confocal microscopy. For animation of Z-stack of optical slices shown in (**a**) see Additional files 10 and 11: Movie 5 and 5a. **b** Astrocyte with misaligned metaphase chromosomes intermingled with RFs (*arrows*). H&E staining. **c** Area in brain stem with many eosin-positive RFs. *Arrow* indicates RF as intranuclear pseudoinclusion. H&E staining. **d** RFs (*arrows*) in astrocyte with several nuclei. (**e**) GFAP immunopositive area (arrow) in the nucleus of an astrocyte. **e1** 2D projection to 3D reconstruction of the cropped area from (**e**). Note that GFAP+ pseudoinclusion (arrow) is located in a deep invagination of nuclear envelope and is not superimposed on the nuclear profile (N). Double immunostaining for GFAP and vimentin, counterstaining with Nissl. Confocal microscopy. **f** Multinuclear astrocyte with RFs. Immunostaining for GFAP and counterstaining with DAPI and Nissl. Note many RFs in vicinity of micronuclei in F’. Confocal microscopy. Scale bars: 8 μm in (**a**); 6 μm in (**b**, **d**); 12 μm in (**c**); 4 μm in (**e**); 12 μm in (**e1**); 11 μm in (**f**)

The prominent feature of the astrocytes with lobulated and large nuclei in the KI homozygous mice should be noted. They often contained GFAP+ spots in the nuclei that looked like intranuclear inclusions (Fig. 7b1). These were extremely rare in TG and double mutant mice. Analysis of the 3D structure of such nuclei with confocal microscopy and of their ultrastructure revealed that these GFAP+’inclusions’ were RFs located in deep cytoplasmic invaginations of nuclei (Fig. 7b, c), hence “pseudoinclusions”.

We have proposed [28] that nuclear changes in abnormal astrocytes might be caused by arrested mitoses. To look for any spatial relationships between RFs and chromosomes during mitosis, we examined the hippocampi of 1 week-old double mutant mice, which show high astrocyte mitotic activity [28]. We found that RFs in mitotic astrocytes were located between metaphase chromosomes (Fig. 8a; Additional files 10, 11 and 12: Movie 5, 5a, 5b). This localization of RFs suggested that the inclusions caused chromosome misalignment, possibly resulting in mitotic arrest. We examined astrocytes with Ki67+ nuclei that appeared abnormal (highly undulated or with several micronuclei). These astrocytes also contained large numbers of RFs (Additional file 13: Figure S5; Additional files 14 and 15: Movie 6 and 6a). Based on all of these observations we conclude that RFs may participate in abnormal chromosomal segregation and cause mitotic abnormalities and/or mitotic arrest.


Additional file 10: Movie 5. Animation of Z stack of optical slices in Fig. 8a (shown only GFAP and DAPI). (AVI 5304 kb)



Additional file 11: Movie 5a. Animation of Z stack of optical slices in Fig. 8a (only DAPI shown) 9a (shown only DAPI). (AVI 4293 kb)



Additional file 12: Movie 5b. (AVI 4315 kb)



Additional file 14: Movie 6. Animation of Z stack of optical slices of Additional file 13: Figure S5a1. (AVI 4380 kb)



Additional file 15: Movie 6a. Animation of Z stack of optical slices of Additional file 13: Figure S5b1. (MOV 4661 kb)


We examined autopsy brain tissues from two patients with AxD and found nuclear pathology similar to that in mouse astrocytes. Many astrocytes had loosely-distributed metaphase chromosomes (corresponding to arrested in metaphase mitoses) intermingled with RF in enlarged bodies (Fig. 8b). Some astrocytes had nuclei with pseudoinclusions, i.e. eosinophilic and GFAP+ material of RFs within nuclei (Fig. 8c and e). Multinucleated astrocytes with several nuclei and RFs were also common findings (Fig. 8d and f).

Many astrocytes in every strain showed enlarged cell bodies and nuclei, which may indicate polyploidy [24]. To assess this possibility we examined the numbers of centrosomes and found multiple centrosomes per cell, indicating that these astrocytes had undergone mitosis without cytokinesis (Additional file 16: Figure S6). We have previously shown multiple centrosomes in abnormal astrocytes in the double mutant mice [28].

## Discussion

In this study we examined four different strains of AxD mutant mice at three different ages to observe stages of RF formation. At all times and in all lines of the mice we found a high degree of variability in the sizes and shapes of RFs. The appearance of RFs ranged from large, dense structures to small, less dense structures that appeared to be deposited on intermediate filaments. We also found that DAPI was an excellent marker for RFs, allowing us to view the distributions of RFs.

### How do RFs form?

**Fig. 9:**
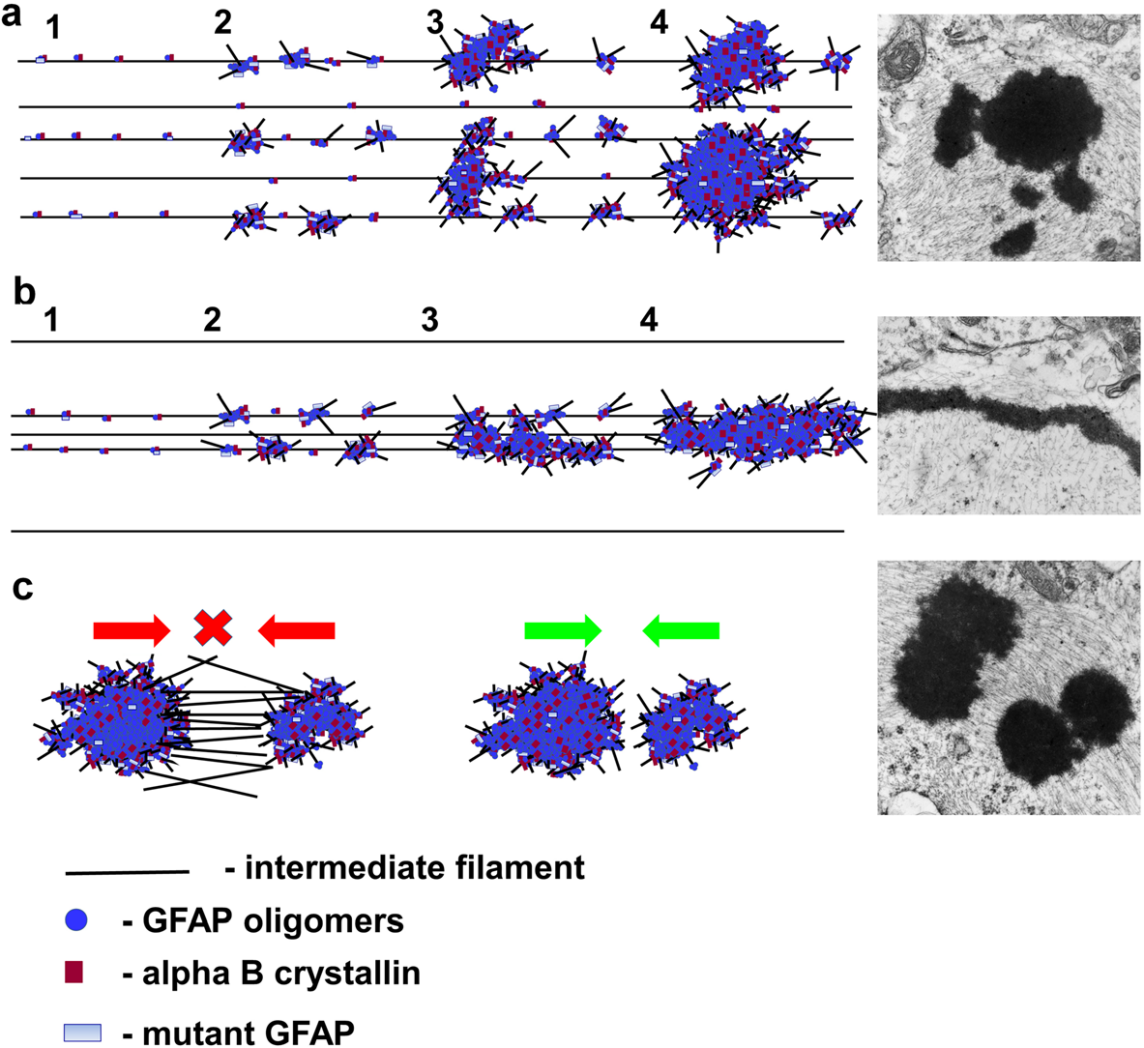
Schematic presentation of RF formation and growth. **a** RFs begin to form as small aggregates of GFAP oligomers with alphaB-crystallin on intermediate filaments. Subsequent accumulation of oligomers with/or without alphaB- crystallin and possibly other components of RFs causes enlargement of RFs. The growth of RFs may proceed with variable speed, generating RFs of different sizes. This type of RF growth is predominant and gives rise to oval RFs. It was observed in every line of AxD mice. **b** Elongated RFs are more typical for KI mice are formed in areas with a relative paucity of intermediate filaments. Aggregation of GFAP oligomers and additional proteins (as in A) occurs along the length of filaments. **c** Some large RFs may be formed by a ‘fusion’ of neighboring RFs of moderate size (*Green*). GFAP filaments linking RFs may prevent RF fusion and preserve RFs as isolated units (*Red*)

We found many examples in which small RFs were connected to adjacent, larger RFs by filamentous and/or granular material, suggesting that RFs may form by the continued incorporation of small RFs into the larger inclusions. The smaller RFs were less electron-dense and in many we found structural heterogeneity. Intermediate filaments coursed through the small inclusions, suggesting that an early stage of RF formation is the deposition of electron dense material on filaments. We also observed in the small RFs a granular, electron-dense material of a relatively homogeneous size, about 15-30 nm. We found similar granular material attached to filaments outside the RFs, suggesting that small deposits on filaments represent an even earlier stage. These observations are consistent with a model in which the deposition of electron dense material on filaments is followed by more deposition and growth of the inclusions, followed by aggregation of small inclusions or incorporation into larger RFs. The deposited material likely attracts more material to the growing aggregate. Figure 9 depicts a possible scheme of RF formation.

Immuno-electron microscopy showed that the granular electron-dense material contains both alphaB-crystallin and GFAP. We did not look for other RF proteins in the smallest aggregates, however, and other proteins may be there as well and may be important in RF formation. Nevertheless, since alphaB-crystallin binds to GFAP [32], these two proteins could be sufficient to form aggregates. The small, granular material attached to filaments contains GFAP and alphaB-crystallin [8]. Thus, one possible mechanism underlying RF formation is the binding of alphaB-crystallin to wild type and/or mutant GFAP monomers or oligomers, which then can associate with filaments. Aggregation into RFs is likely to be a phenomenon dependent upon protein concentration and the stoichiometry of GFAP and alphaB-crystallin levels. We previously found that overexpressing alphaB-crystallin in cultured astrocytes with normal GFAP levels debundled intermediate filaments, but did not depolymerize them or lead to the formation of RFs [8].

However, genetically overexpressing alphaB-crystallin in astrocytes in AxD mice reduced the oxidative stress response, RFs, seizures, and restored astrocyte glutamate uptake ([6]). We do not know the stoichiometry of GFAP and alphaB-crystallin in individual astrocytes in either the AxD mice or in the AxD mice that overexpressed alphaB-crystallin. The alphaB-crystallin transgenics produce more alphaB-crystallin from the outset, a state somewhat different from AxD itself, and which might alter RF formation from the beginning.

Whether proteolytic degradation of GFAP plays a role in RF formation is not known. We previously performed Western blots of mouse AxD model brains and observed only a small band of GFAP- immunoreactive material migrating more rapidly than the main protein band, indicating a minimum level of GFAP proteolysis. Western blots, however, do show a series of less rapidly migrating bands, which are likely to represent ubiquitinated GFAP and GFAP oligomers [32–34]. However, Chen et al. [2] present evidence that GFAP is a substrate for caspase 6 and using an antibody to the cleaved peptide, found proteolyzed GFAP in cultured cells that expressed the R239H GFAP mutation and in the heterozygous KI mice. The small, 26 kDa fragment is largely insoluble, using a deoxycholate and Triton X-100 buffer, and thus it is possible that this fragment might be found in RFs and contribute to their formation.

### Mouse RFs appear identical to human RFs

The idea that RFs are formed from an accumulation of smaller inclusions is consistent with reports of human biopsy and autopsy material. Thus, EM reports of human AxD note small RF-like material at the borders of larger RFs [1, 10, 19, 23]. Borrett and Becker (1985) reported a brain biopsy from a 14 week-old infant, which showed many small RFs in a bed of intermediate filaments, and concluded that these inclusions represented early RFs. We have observed the same small RFs in many of the mouse astrocytes. A biopsy of a 34 month-old child, described in the same report, showed a large RF and also several smaller ones within the large skein of filaments (see Fig. 6 in [1]). Variation in the size of RFs within a single astrocyte was also reported by Schochet et al. [23], who reported ultrastructural findings in an 8 month-old child showing a spectrum of sizes of RFs, including many small ones. RFs were connected to each other by filaments and granular material. Astrocytes in adult forms of AxD also show variation in the sizes of RFs with small inclusions situated among the larger ones [25, 29]. Neuropathologists have not previously commented on the small, granular profiles or the less dense matrix of small RFs, although Herndon et al. [10] commented on the “thickening” of filaments in the vicinity of a RF (their Fig. 13), and granular deposits on the filaments. Of course, nothing was known about the molecular composition of RFs at that time.

### RFs can form anywhere in the astrocyte and continue to form over time

The presence of small RFs in all parts of the astrocyte, including cell body, processes, and endfeet, suggests that RFs can be independently formed throughout the cell, although we cannot rule out the possibility that some form in the cell body and are then transported to endfeet.

RFs continue to be generated over time in parallel with the accumulation of GFAP. We observed the small, electron dense aggregates in 1 month-old as well as in 1 year-old AxD mice. Although at every timepoint there was a large degree of variability in RF size, the numbers and sizes of RFs were greater in the older mice. Thus, RF formation is an ongoing process in AxD, rather than just occurring at early stages of the disorder.

However, even in the areas with high levels of astrogliosis not every astrocyte contained RFs. We were surprised that some astrocytes filled with GFAP did not contain RFs, whereas nearby astrocytes contained many RFs. Different levels of alphaB-crystallin and/or some other heat shock proteins that participate in RF formation might be responsible for the variable numbers of RFs in astrocytes. We could not exclude the possibility, however, that the very small RFs (200–300 nm in size) seen with the electron microscope are below the resolution of the confocal microscope.

### Do RFs influence astrocyte phenotype?

Do RFs influence astrocyte phenotype or are they innocuous structures that have no effects on cell morphologies or functions? Given that RFs are always located within large skeins of intermediate filaments, it is difficult to dissect clearly the effects of RFs per se from those of the filament accumulations. Indeed, some changes of astrocytes, such as the new expression of CD44, do not depend on the presence of RFs, since high levels of CD44 were observed in cells with and without RFs. A similar case is true for other markers of astrocytes that we used (GLT-1, GLAST, ferritin, Kir 4.1, vimentin, nestin, not shown).

The two prominent changes that were consistently associated with large numbers of RFs were 1) a thickening and shortening of main processes and a loss of miniature leaf-like processes, and 2) abnormalities in nuclear morphologies. Astrocytes retract many of their processes during arrested mitoses and then extend them to their normal size after slippage from mitotic arrest (manuscript in preparation). Astrocytes filled with RFs may not be able to restore the normal shapes and sizes of their processes, presumably due to a disruption of proper cytoskeletal orientation by RFs. A loss of small, distal processes may have special significance because distal processes that isolate synapses are responsible for extraneuronal ion and transmitter homeostasis. Their absence might severely alter neuronal excitability [12, 18]. Note that the double mutant AxD mice develop seizures at 4-5 weeks of age. Transgenic and KI AxD mice are more susceptible to kainic acid-induced seizures compared to wild type mice [7, 31]. Those astrocytes that displayed such changes also showed enlargement and irregular forms of nuclei. We consider that the nuclear abnormalities originate due to arrested mitosis when RFs interfere with chromosome congression into the metaphase plate and subsequent segregation into two daughter groups, not allowing cells to fulfill cytokinesis. Finding RFs in mitotic astrocytes in mouse and in human indicates that at least some RFs do not depolymerize during mitosis. RFs between chromosomes would likely interfere with normal chromosome segregation and spindle formation. In addition, the formation of the nuclear envelope in telophase may also be influenced by RFs. Filaments are bound by the cytoskeleton linker protein plectin to nesprin-3, located in outer leaflet of the nuclear envelope [27]. Plectin accumulates in RFs [35], and thus could link RFs to the nuclear envelope and interfere with the formation or dissolution of the nuclear envelope.

We also observed that RFs with associated filament bundles often excluded membranous organelles, segregating those organelles either to a paranuclear position or to the periphery of the cell. In cultures of AxD astrocytes, we found separation of Golgi and ER complexes and fragmentation of the Golgi apparatus by large bundles of filaments and RFs (Guilfoyle and Sosunov, unpublished). These observations suggest that membrane trafficking may be disrupted in AxD astrocytes. RFs may increase the mechanical stability of filament bundles/aggregates and thus create mechanical barriers for intracellular trafficking as well for chromosome congression and segregation during mitosis.

### DAPI as a new method for visualizing RFs

We found that DAPI can be used as a reliable and reproducible method of visualizing RFs. An advantage of this method is the ability to combine it with routine immunohistochemical procedures. Why RFs are stained with DAPI is not clear. One possible explanation may be that DAPI, like FJB, has an affinity for highly acidic structures. Fluorescent Nissl Stain (Neuro Trace, Molecular Probes) also gives positive staining of RFs (unpublished results) but in comparison with DAPI is much less reproducible and does not stain the small puncta-like RFs.

## Conclusions

1. RFs appear to originate as small, osmiophilic masses containing both GFAP and alphaB-crystallin deposited on bundles of intermediate filaments.
2. RFs continue to form within AxD astrocytes over time.
3. DAPI is a reliable marker for RFs and can be used with immunolabeling.
4. RFs and bundles of filaments appear to interfere with the successful completion of astrocyte mitosis.

## Additional files

Additional file 1: Figure S1. Representative ultrastructure of RFs in astrocytes in KI (**a**, **c**) and TG (**b**, **d**) AxD mice. Note that astrocytes in KI mice often have many polymorphic RFs located in a local area in the cell body (**a**) whereas in TG mice RFs are distributed all over the perikaryon (**b**). Undulating, ramified RFs (**c, c1**) predominated in KI mice, in TG mice oval RFs (**d, d1**) are typical type. Scale bars: 2 μm in **a**, **b**; 6 μm in **c**, **d**. (JPG 2341 kb)

Additional file 2: Figure S4. Heterogeneity in astrocyte features and RFs shapes in AxD mice. **a**) Subpial area in the neocortex in 1 year-old TG mouse. Note that astrocytes marked with arrows contain many RFs whereas neighboring astrocytes (asterisk) with high levels of GFAP do not contain RFs. Immunostaining for GFAP, counterstaining with DAPI. Confocal microscopy. **b**) Variability in the shapes of RFs stained with FJB in neocortical astrocytes in 1 year-old TG mouse. **b1**) and **b2**) enlarged boxed areas in (**b**) show neighboring astrocytes filled with ball-like (**b1**) and elongated (**b2**) RFs. Confocal microscopy. **c**) EM of neighboring astrocytes (1 and 2) filled with RFs of different shape and size. 1 month-old double mutant mouse.N- astrocyte nuclei. Scale bars: 75 μm in **a**; 60 μm in **b**; 2 μm in **c**. (JPG 1244 kb)

Additional file 3: Figure S2. Different pattern of GFAP immunostaining in KI homozygous (**a**, **d**) in comparison with KI heterozygous (**b**, **e**) and TG (**c**, **f**) mice. Note that in homozygous KI mice GFAP immunostaining has punctate pattern and delineates oval profiles with bright fluorescence whereas in KI heterozygous and TG mice GFAP immunostaining of astrocytes has a homogeneous pattern. Confocal microcopy. Scale bars: 60 μm in **a**-**c**; 20 μm in **d**-**f**. (JPG 773 kb)

Additional file 4: Figure S3. Rosenthal fibers (RFs) in subpial area in the neocortex of a 1 year-old KI homozygous mouse (**a**) and in human AxD brain (**b**). **a1**) Single optical slice from the enlarged boxed area in **a**. Note many small oval profiles positive for GFAP with central part stained with DAPI (arrows, only some marked). Immunostaining for GFAP, counterstaining with DAPI. Confocal microscopy. **b1**) and **b2**) enlarged boxed areas in **b** show DAPI staining of RFs in astrocytes. Note that large astrocyte profiles are filled with many densely packed RFs. Double immunostaining for GFAP and vimentin (VIM). Counterstaining with DAPI. Confocal microscopy. Scale bars: 80 μm. (JPG 2054 kb)

Additional file 13: Figure S5. RFs in astrocytes, which have just completed mitosis, with several lobulated Ki67+ nuclei in 1 week-old double mutant mouse.Animation of Z-stack of optical slices are shown in Additional file 14: Movies 6 (for image **a1**) and in Additional file 15: movie 6a (for image **b1**). Double immunostaining for GFAP and Ki67, counterstaining with DAPI. Confocal microscopy. Black and white images show DAPI staining. Scale bars: 12 μm. (JPG 589 kb)

Additional file 16: Figure S6. Multiplication of centrosomes in large polyploidal astrocytes in 1 year old KI homozygous mice. **a**, **b**) Extra centrioles (arrows) identified with pericentrin in astrocytes with large, lobulated nuclei. Note highly lobulated irregular shapes of nuclei in **a’** and **b’**. Double immunostaining for GFAP and pericentrin, counterstaining with Nissl. Confocal microscopy. **c**) Ultrastructure of the astrocyte with several nuclear profiles (N) indicating lobulated nucleus and with two pair of centrioles (asterisk and star in **c2**). Note highly lobulated nuclear profiles (N) in **c1**. Arrow in **c1** indicates a RF. Electron microscopy. **c1** and **c2** enlarged boxed areas in **c**. Scale bars: 20 μm in **a**; 8 μm in **b**; 5 μm in **c**. (JPG 1181 kb)

## Abbreviations

AxD: Alexander disease
FJB: Fluoro jade B
GFAP: Glial fibrillary acidic protein
KI: Knock-in
RF: Rosenthal fiber
Tg: Transgenic
