# Supplementary figures and images for: The origin of Rosenthal fibers and their contributions to astrocyte pathology in Alexander disease
Source: Acta Neuropathol Commun. 2017 Mar 31;5:27. doi: 10.1186/s40478-017-0425-9 (PMC5374671; doi:10.1186/s40478-017-0425-9)

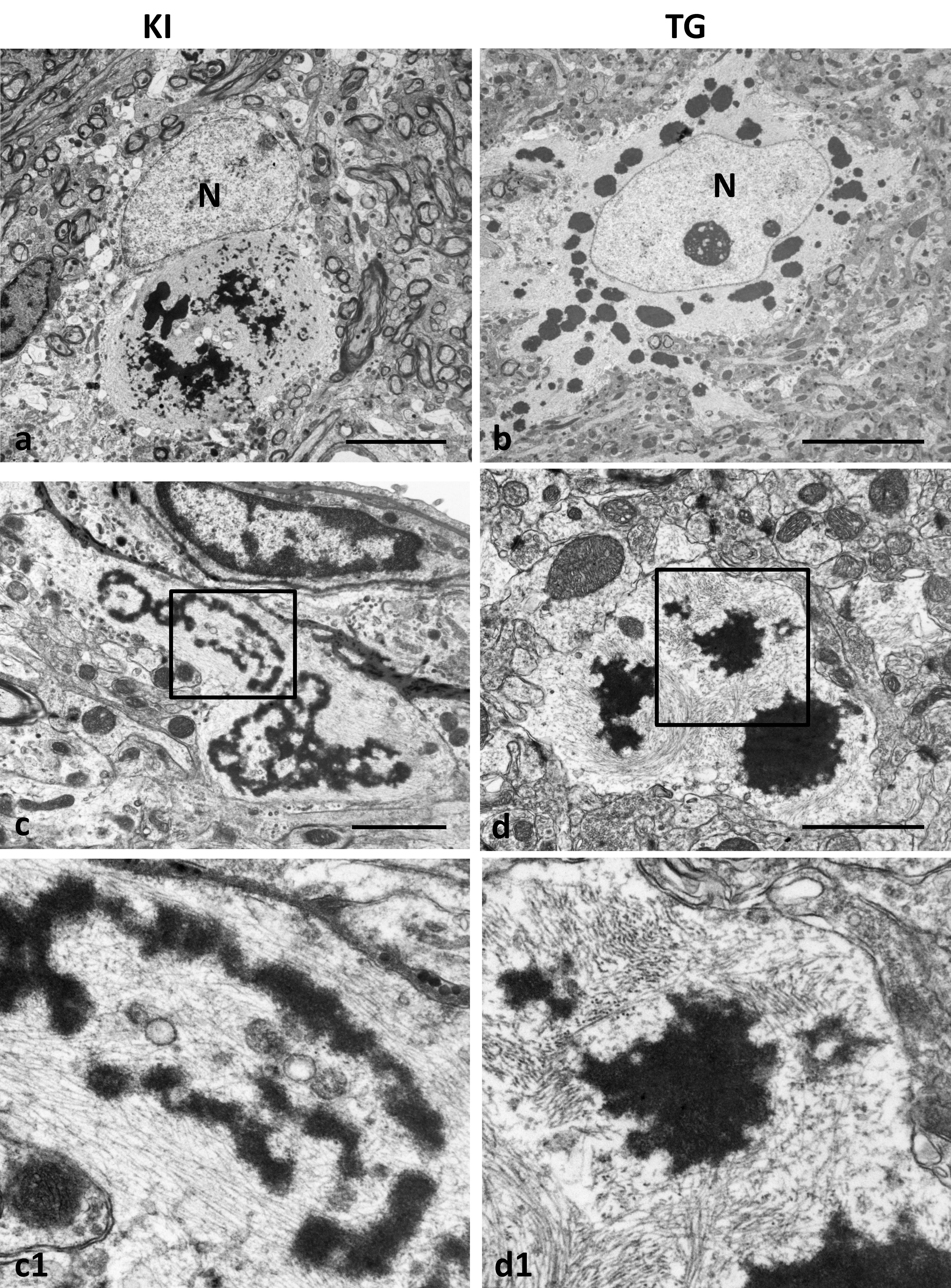

Supplement: Supplementary file 11 — Representative ultrastructure of RFs in astrocytes in KI (a, c) and TG (b, d) AxD mice. Note that astrocytes in KI mice often have many polymorphic RFs located in a local area in the cell body (a) whereas in TG mice RFs are distributed all over the perikaryon (b). Undulating, ramified RFs (c, c1) predominated in KI mice, in TG mice oval RFs (d, d1) are typical type. Scale bars: 2 μm in a, b; 6 μm in c, d. (JPG 2341 kb) [file 40478_2017_425_MOESM1_ESM.jpg]

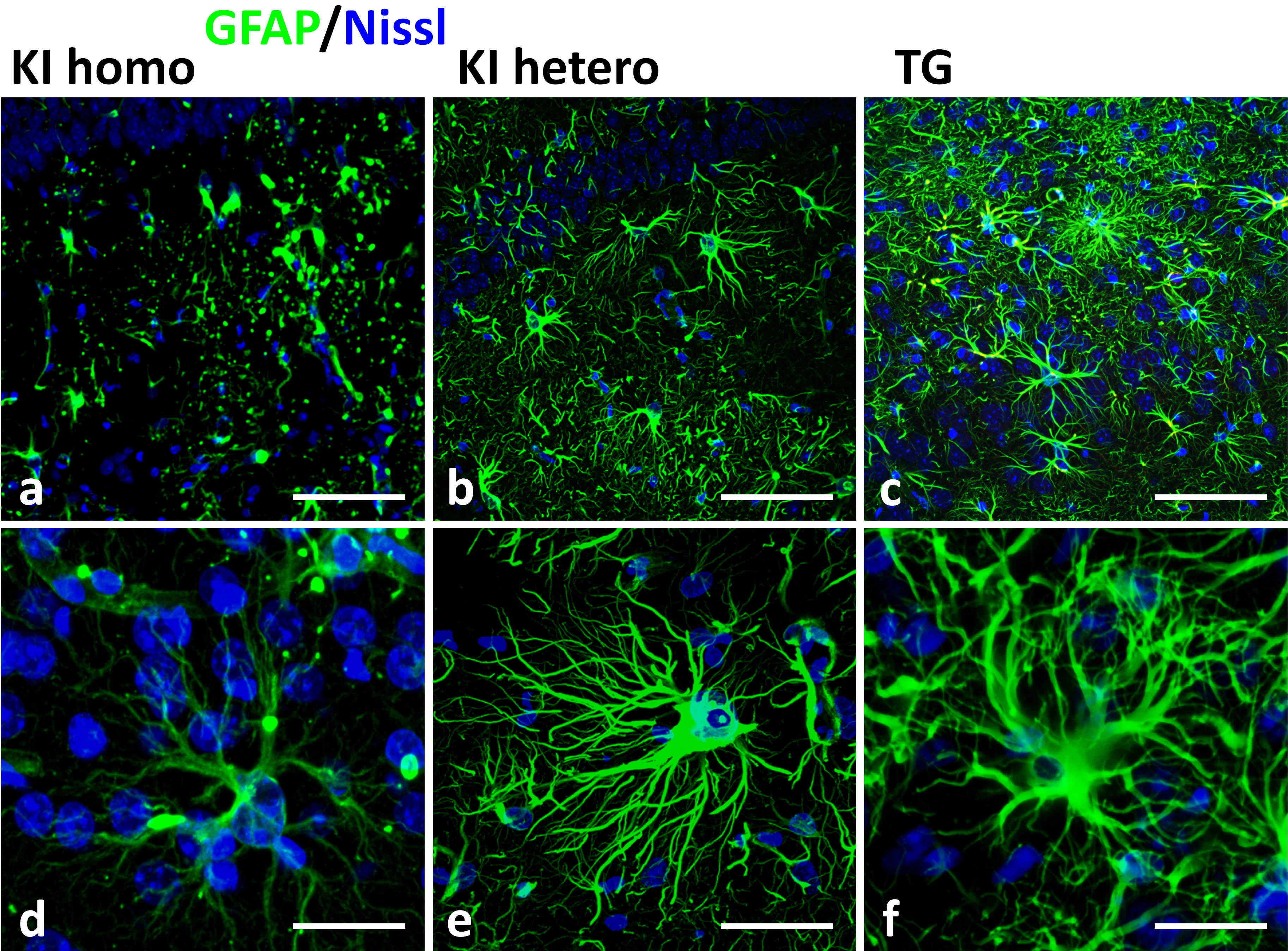

Supplement: Supplementary file 12 — Heterogeneity in astrocyte features and RFs shapes in AxD mice. a) Subpial area in the neocortex in 1 year-old TG mouse. Note that astrocytes marked with arrows contain many RFs whereas neighboring astrocytes (asterisk) with high levels of GFAP do not contain RFs. Immunostaining for GFAP, counterstaining with DAPI. Confocal microscopy. b) Variability in the shapes of RFs stained with FJB in neocortical astrocytes in 1 year-old TG mouse. b1) and b2) enlarged boxed areas in (b) show neighboring astrocytes filled with ball-like (b1) and elongated (b2) RFs. Confocal microscopy. c) EM of neighboring astrocytes (1 and 2) filled with RFs of different shape and size. 1 month-old double mutant mouse.N- astrocyte nuclei. Scale bars: 75 μm in a; 60 μm in b; 2 μm in c. (JPG 1244 kb) [file 40478_2017_425_MOESM2_ESM.jpg]

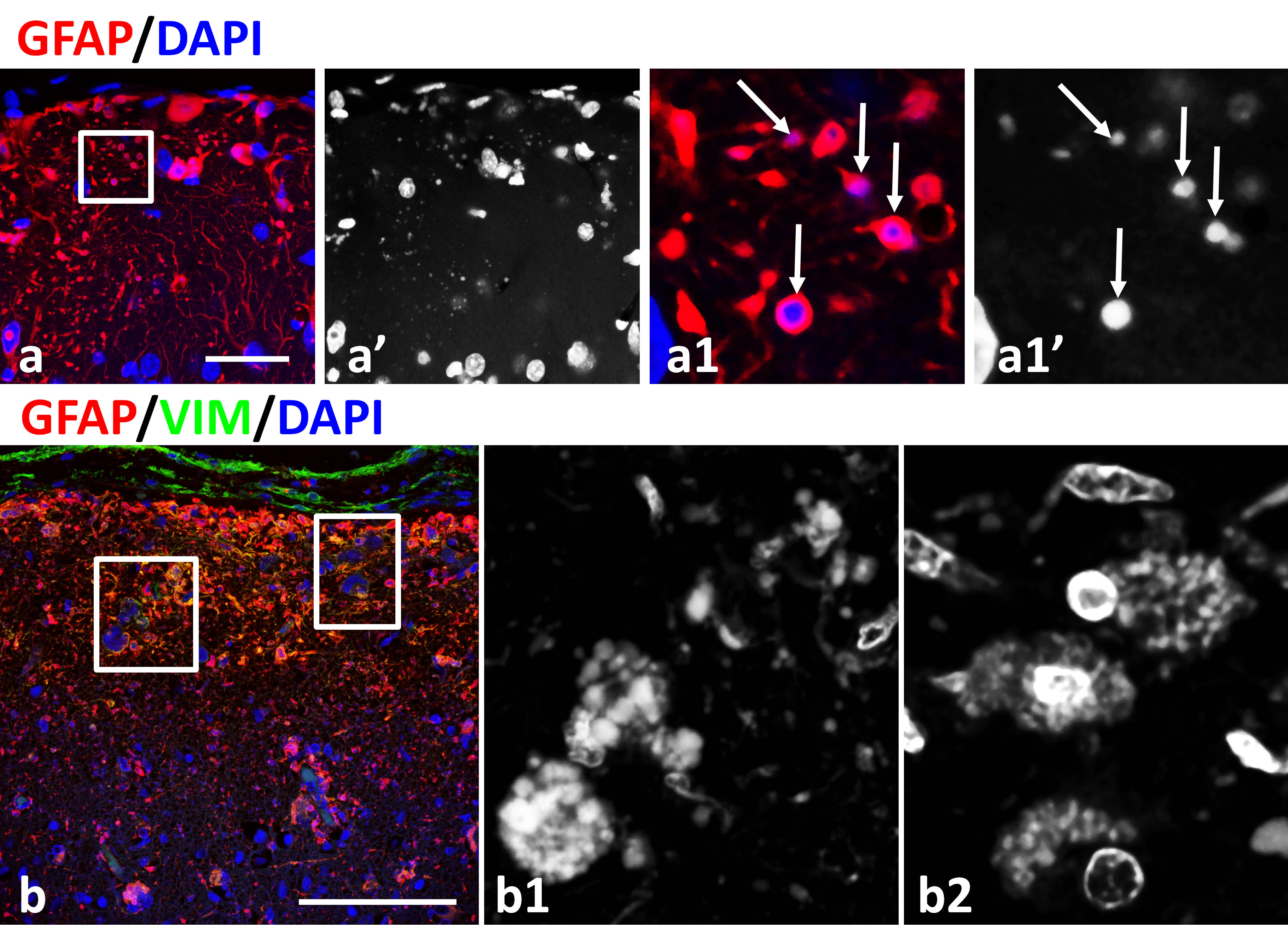

Supplement: Supplementary file 13 — Different pattern of GFAP immunostaining in KI homozygous (a, d) in comparison with KI heterozygous (b, e) and TG (c, f) mice. Note that in homozygous KI mice GFAP immunostaining has punctate pattern and delineates oval profiles with bright fluorescence whereas in KI heterozygous and TG mice GFAP immunostaining of astrocytes has a homogeneous pattern. Confocal microcopy. Scale bars: 60 μm in a-c; 20 μm in d-f. (JPG 773 kb) [file 40478_2017_425_MOESM3_ESM.jpg]

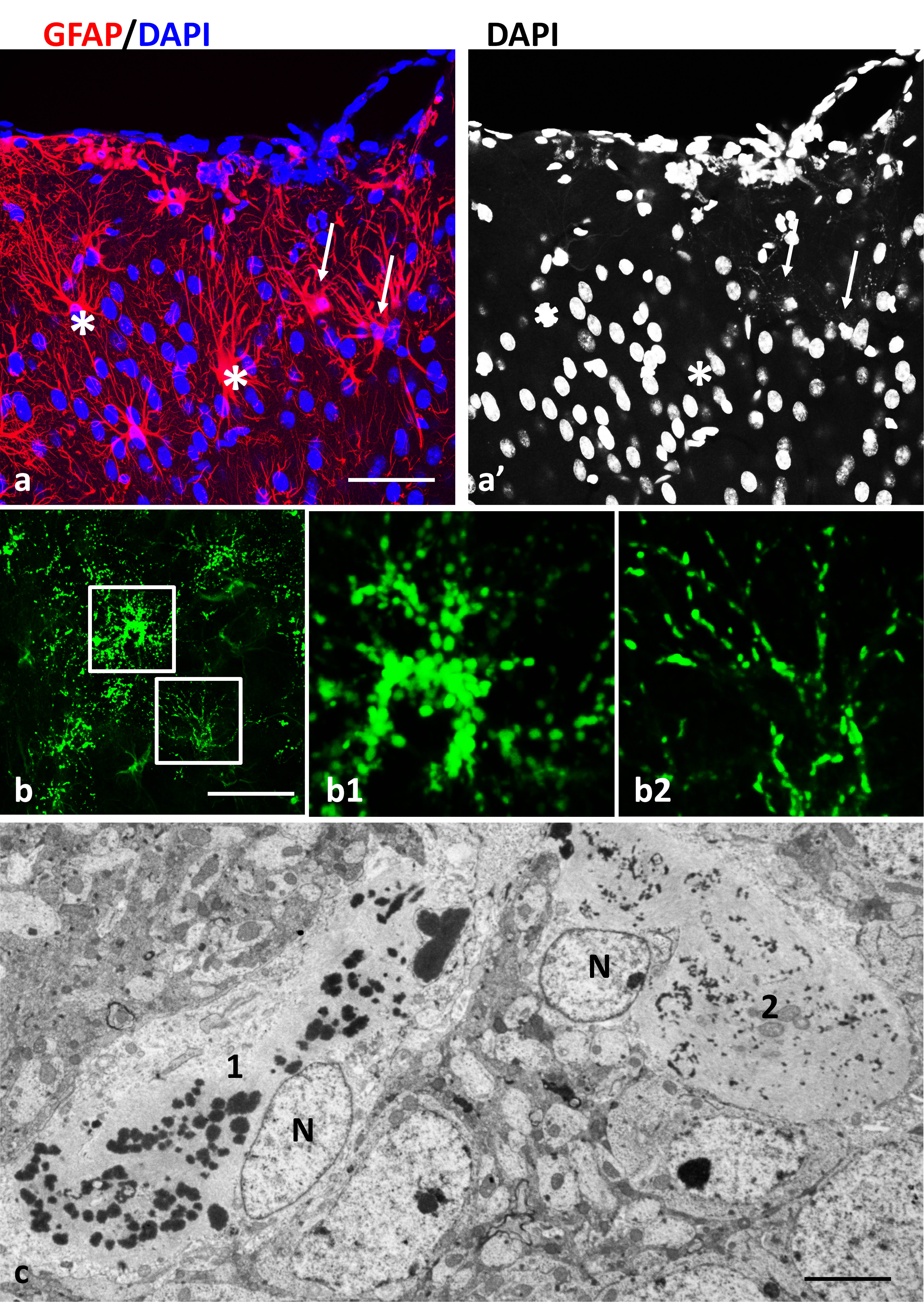

Supplement: Supplementary file 14 — Rosenthal fibers (RFs) in subpial area in the neocortex of a 1 year-old KI homozygous mouse (a) and in human AxD brain (b). a1) Single optical slice from the enlarged boxed area in a. Note many small oval profiles positive for GFAP with central part stained with DAPI (arrows, only some marked). Immunostaining for GFAP, counterstaining with DAPI. Confocal microscopy. b1) and b2) enlarged boxed areas in b show DAPI staining of RFs in astrocytes. Note that large astrocyte profiles are filled with many densely packed RFs. Double immunostaining for GFAP and vimentin (VIM). Counterstaining with DAPI. Confocal microscopy. Scale bars: 80 μm. (JPG 2054 kb) [file 40478_2017_425_MOESM4_ESM.jpg]

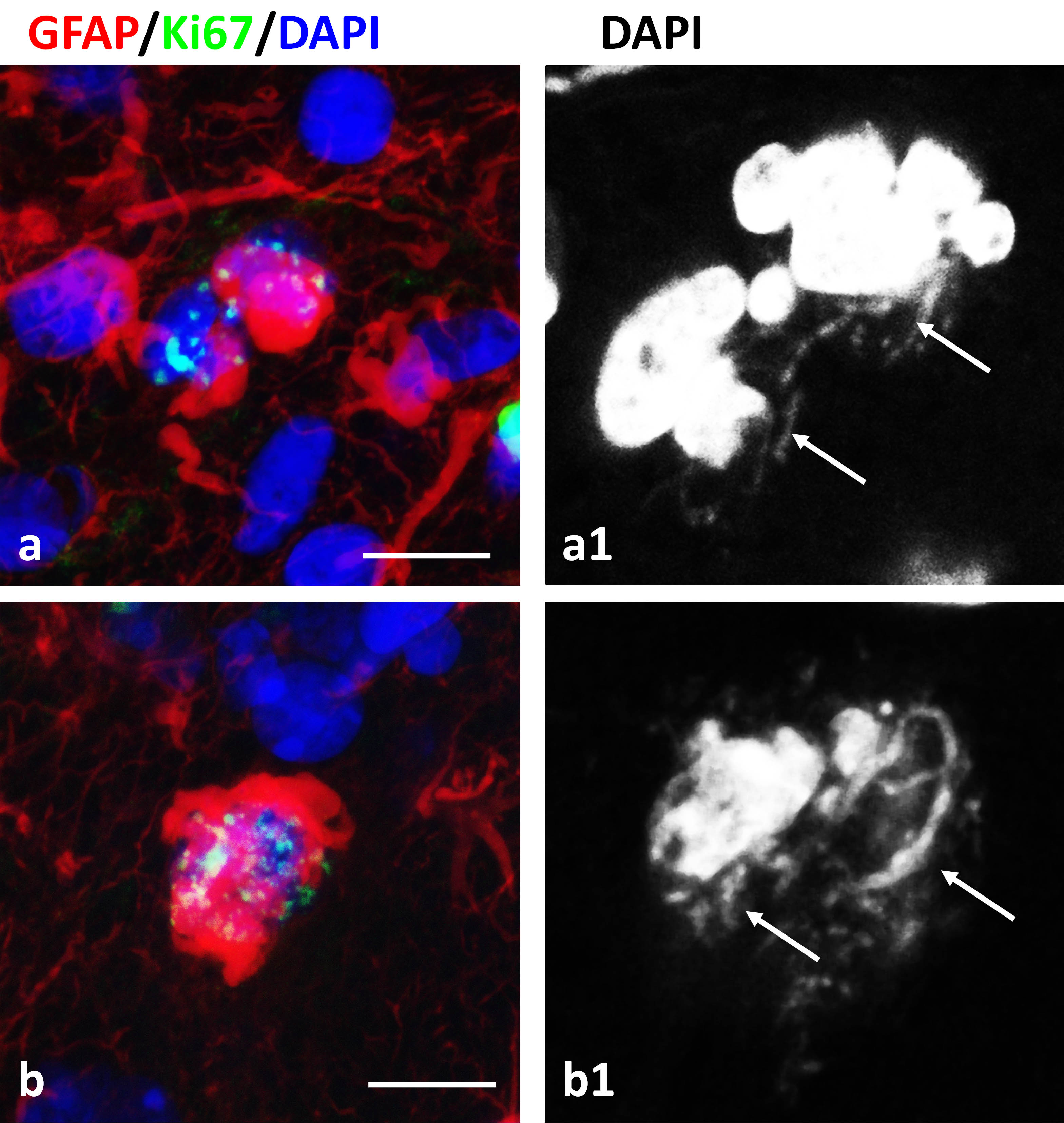

Supplement: Supplementary file 15 — RFs in astrocytes, which have just completed mitosis, with several lobulated Ki67+ nuclei in 1 week-old double mutant mouse.Animation of Z-stack of optical slices are shown in Additional file 14: Movies 6 (for image a1) and in Additional file 15: movie 6a (for image b1). Double immunostaining for GFAP and Ki67, counterstaining with DAPI. Confocal microscopy. Black and white images show DAPI staining. Scale bars: 12 μm. (JPG 589 kb) [file 40478_2017_425_MOESM13_ESM.jpg]

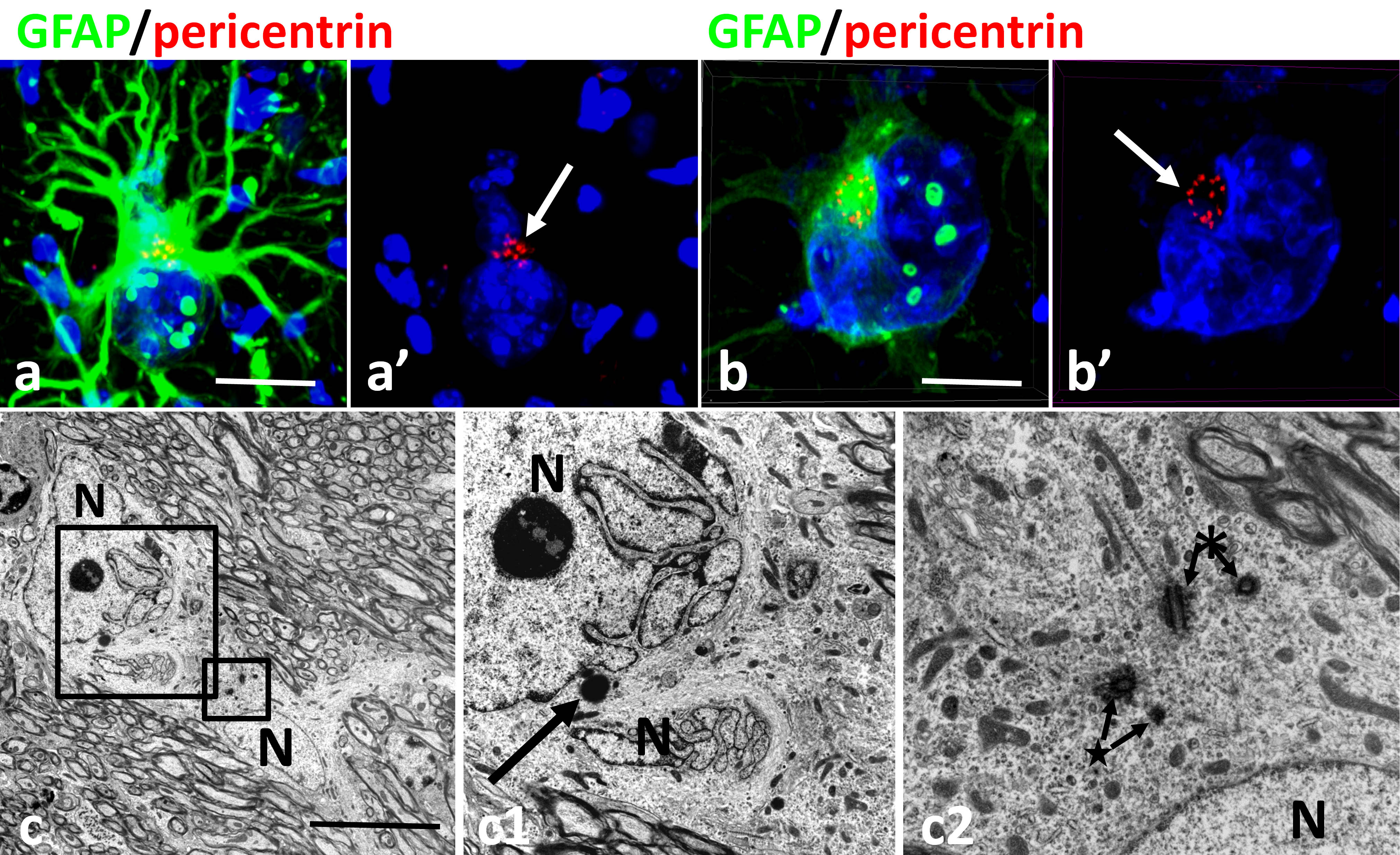

Supplement: Supplementary file 16 — Multiplication of centrosomes in large polyploidal astrocytes in 1 year old KI homozygous mice. a, b) Extra centrioles (arrows) identified with pericentrin in astrocytes with large, lobulated nuclei. Note highly lobulated irregular shapes of nuclei in a’ and b’. Double immunostaining for GFAP and pericentrin, counterstaining with Nissl. Confocal microscopy. c) Ultrastructure of the astrocyte with several nuclear profiles (N) indicating lobulated nucleus and with two pair of centrioles (asterisk and star in c2). Note highly lobulated nuclear profiles (N) in c1. Arrow in c1 indicates a RF. Electron microscopy. c1 and c2 enlarged boxed areas in c. Scale bars: 20 μm in a; 8 μm in b; 5 μm in c. (JPG 1181 kb) [file 40478_2017_425_MOESM16_ESM.jpg]
